# Herb network construction and co-module analysis for uncovering the combination rule of traditional Chinese herbal formulae

**DOI:** 10.1186/1471-2105-11-S11-S6

**Published:** 2010-12-14

**Authors:** Shao Li, Bo Zhang, Duo Jiang, Yingying Wei, Ningbo Zhang

**Affiliations:** 1 MOE Key Laboratory of Bioinformatics and Bioinformatics Division, TNLIST / Department of Automation, Tsinghua University, Beijing, China

## Abstract

**Background:**

Traditional Chinese Medicine (TCM) is characterized by the wide use of herbal formulae, which are capable of systematically treating diseases determined by interactions among various herbs. However, the combination rule of TCM herbal formulae remains a mystery due to the lack of appropriate methods.

**Methods:**

From a network perspective, we established a method called Distance-based Mutual Information Model (DMIM) to identify useful relationships among herbs in numerous herbal formulae. DMIM combines mutual information entropy and “between-herb-distance” to score herb interactions and construct herb network. To evaluate the efficacy of the DMIM-extracted herb network, we conducted *in vitro* assays to measure the activities of strongly connected herbs and herb pairs. Moreover, using the networked *Liu-wei-di-huang* (LWDH) formula as an example, we proposed a novel concept of “co-module” across herb-biomolecule-disease multilayer networks to explore the potential combination mechanism of herbal formulae.

**Results:**

DMIM, when used for retrieving herb pairs, achieves a good balance among the herb’s frequency, independence, and distance in herbal formulae. A herb network constructed by DMIM from 3865 Collaterals-related herbal formulae can not only nicely recover traditionally-defined herb pairs and formulae, but also generate novel anti-angiogenic herb ingredients (e.g. Vitexicarpin with IC50=3.2 μM, and Timosaponin A-III with IC50=3.4 μM) as well as herb pairs with synergistic or antagonistic effects. Based on gene and phenotype information associated with both LWDH herbs and LWDH-treated diseases, we found that LWDH-treated diseases show high phenotype similarity and identified certain “co-modules” enriched in cancer pathways and neuro-endocrine-immune pathways, which may be responsible for the action of treating different diseases by the same LWDH formula.

**Conclusions:**

DMIM is a powerful method to identify the combination rule of herbal formulae and lead to new discoveries. We also provide the first evidence that the co-module across multilayer networks may underlie the combination mechanism of herbal formulae and demonstrate the potential of network biology approaches in the studies of TCM.

## Background

Traditional Chinese Medicine (TCM) is an important part of the current medical system. It aims to restore the whole-body balance in patients by using herbal formula (*Fang-Ji* in Mandarin), which is usually composed of two or more medicinal herbs and has the capacity of systematically treating disease [1]. Naturally occurring herbs and herbal ingredients organized into certain formula have been shown to have potential interaction effects. These include mutual enhancement, mutual assistance, mutual restraint and mutual antagonism [2,3]. For example, synergistic interactions occur when the efficacy of combinations of herbs (or ingredients) is greater than the summed responses of each individual herb or ingredient. Adams *et al*. [4] recently reported the synergistic, additive and antagonistic effects exerted by different combinations of six herbal extracts on the viability of prostate cancer cell lines. Wang reported that a *Realgar*-*Indigo naturalis* formula is beneficial for the treatment of promyelocytic leukemia; the synergistic effects exerted by several components of this formula are well documented [5]. Ung *et al*. [6] conducted an analysis of 394 TCM herb pairs and 2470 non-TCM herb pairs using artificial intelligence methods and considering four classes of herbal properties as features including character, taste, meridian, and toxicity level. Their study revealed that herb pairs in TCM contain features distinguishable from those of non-TCM herb pairs. Schmidt *et al.*[7] believed that mixtures of interacting compounds produced by plants may become a valuable asset and an important resource for drug discovery, especially for the development of combinational therapeutics.

However, there is still a lack of appropriate methods to learn how and why many herbs are grouped in certain formulae, and the combination rule embedding numerous herbal formulae remain unknown. Traditionally herbs have taken different roles in a typical herbal formula; they are usually expressed in the organization order as Master, Adviser, Soldier and Guide (MASG), each of which is given certain natural properties including Cold, Cool, Neutral, Warm or Hot. Understanding the combination rule of herbal formulae will not only benefit the modernization of TCM but may also be helpful for the way drugs are studied. A good example of the potential of TCM involves angiogenesis. TCM is known to be effective for the treatment of angiogenesis which is the main type of pathological vascular growth associated with various diseases such as cancer and rheumatoid arthritis [8,9]. We know that more than 60% of the current cancer chemotherapeutic agents are natural products or small molecules based on natural product leads [10,11]. Many pro-angiogenic and anti-angiogenic plant components are potentially useful for curing angiogenic disorders and are well tolerated [9]. Especially, herbs originally used for treating “Collaterals (*Luo* in Mandarin) diseases” in TCM have been found to be active on angiogenic disorders [12]. As a consequence, combining traditional herbal formulae with existing biological knowledge might allow researchers to rapidly identify combination treatments for angiogenic disorders.

Recently, a remarkable development has been the use of systems biology, especially network biology, in drug study. This methodology has revealed the systematic mechanisms of complex disease and has highlighted the paradigm shift from “one drug, one target” to “multicomponent therapeutics, biological networks” [13,14]. Even though the scientific community has high expectations for systems pharmacology, this field is still in its infancy because of a poor understanding of cell behaviours and drug-protein interactions. TCM formula is considered to be an empirical system of multicomponent therapeutics which potentially meets the demands of treating a number of complex diseases in an integrated manner [3,14,15]. So, in order to find a relationship between groups of drugs and complex diseases, it is important to introduce a powerful approach to bridge the tradition and the modern, and pursue *a priori* knowledge about the combination rules embedded in TCM. In this work, we developed a Distance-based Mutual Information Model (DMIM) to extract the herb relationships from plentiful herbal formulae. This method was then used to construct the “herb network” from 3865 Collaterals-related herbal formulae, following by *in vitro* experiments designed to evaluate the angiogenic effects and synergistic properties of strongly connected herbs and herb pairs. A new concept of “co-module” was further proposed and network biology analyses were conducted to explore the potential combination mechanism of the networked herbal formulae.

## Methods

### Data sources of herbal formulae

#### Candidate herbal formulae selection

TCM values the “Collaterals” theory and therapy. Using “Collaterals (*Luo*)” as the keyword, we searched the SIRC-TCM Herbal Formula database (http://www.tcm120.com/1w2k/tcm_recipe.asp) which contains 0.14 million herbal formulae. Then we collected 3865 herbal formulae with formula names and functions, or herb’s meridian tropism (*Gui-jing* in Mandarin), or targeted syndromes and diseases containing the keyword. We standardized the herbal formulae by substituting all the polysemes, synonyms and acronyms of the herbs in the dataset using the standardized Herb Name list. The standardized Herb Name list consists of 737 herbs. The 3865 Collaterals-related herbal formulae, as examples, will be subject to the following DMIM analysis.

#### Traditionally-defined herb pairs

The herb pair is the basic unit of a herbal formula. To evaluate the reliability and utility of the DMIM-extracted herb network, 600 traditionally-defined herb pairs recorded in [16] and 301 herb pairs from [17] were collected. This resulted in 775 non-redundant traditionally-defined herb pairs made up of 737 separate herbs in the Collaterals-related herbal formulae.

### Establishment of DMIM Scoring System

#### Numerical representation for herbal formulae

In the DMIM, we turn the normalized formula data into a numeral matrix to indicate the relative position of the herbs in a formula. Assuming there are a total of *n* herbs and *m* formulae, we assign serial numbers to all the herbs from 1 to *n*. As illustrated in Table 1, we use a *m×n* matrix *A* = (*a_ij_*)_m__*__n_ to indicate the formula where the *i*th row vector denotes the components of the *i*th formula, and *a_ij_* is the number of the position of the *j*th herb in *i*th formula ( *a_ij_* =0 means herb *j* is absent in formula *i*). To eliminate the impact of the total number of herbs in one formula, we define the matrix *B*=(*b*_*ij*_) as  where *k* denotes the total number of herbs in a formula.

**Table 1:** Examples for the numerical representation of herbal formulae

Formula	herb_1_	herb_2_	herb_3_	herb_4_	herb_5_	herb_6_	herb_7_	herb_8_	herb_9_	herb_10_
Formula _1_	0	0.5	0	0.25	0	1	0	0	0.75	0

Formula _2_	0.375	0.625	0.875	0.125	0.75	0	1	0.25	0	0.5

Formula _3_	0.5	0	0	0	0	1	0	0	0	0

*Assume there are a total of 10 herbs denoted by herb_1_, herb_2_…herb_10_ (*j*=1, 2…,10), and three herbal formulae containing the following herbs in sequence (*i*=1,2,3): Formula _1_: herb_4_, herb_2_, herb_9_ and herb_6_ (*k*=4); Formula _2_: herb_4_, herb_8_, herb_1_, herb_10_, herb_2_, herb_5_, herb_3_ and herb_7_ (*k*=8); and Formula _3_: herb_1_ and herb_6_ (*k*=2). For herb_2_ in Formula _1_, we calculated that *a_12_* =2 and *b_12_*=2/4=0.5. The matrix representation for this artificial data set is shown in the table.

From this we had matrix *B* , where *b_ij_* indicates the relative position of herb *j* in the formula *i*. Finally, the real data set is represented by a 3865×737 matrix. Then, for given two herbs, *x* and *y*, we deduce that the tendency of *x* and *y* to form a herb pair is dependent on two factors: mutual information entropy characteristics and the average distance between herbs.

#### Mutual information entropy

To begin with, we calculate the traditional mutual information entropy [21] for *x* and *y* as: . Here  is the frequency that herb *x* and herb *y* occurred, and I(x, y,i) is the indicator function of *x* and *y*, showing whether herb *x* and *y* coexist in the formula *i*. is the frequency of herb *x*. It is the same with *P*(*y*). A large value of *MI*(*x*, *y*) indicates a strong correlation between herb *x* and herb *y* .

#### Between-herb-distance

Considering a later order indicates a less importance in the organization of Master, Adviser, Soldier or Guide herbs in a herbal formula, we assume that the further the distance between two herbs in a formula, the less likely they are to be relevant to one another. The distance between herb *x* and herb *y* in the *i*th formula, called the “between-herb-distance”, is defined as: *d*(*x*, *y*, *i*) = |*B*(*x*,*i*) - *B*(*y*, *i*)|. The average distance of herb *x* and herb *y* in the dataset is .

### DMIM scoring system

The DMIM combines the mutual information (*MI*) entropy characteristics and the average distance between herbs (*d*) to form a scoring system, , which describes the tendency of herb *x* and herb *y* to form a herb pair. So when two herb pairs share the same information entropy, the one with the smaller average distance shows a stronger connection. When two herb pairs have the same average distance, the one with the larger information entropy shows a greater interaction.

### Evaluation of the DMIM-extracted herb network

#### *In vitro* assays for evaluating angiogenic activities of DMIM-extracted herbs

We selected major herbal ingredients from DMIM outputs to evaluate angiogenic activities. Two kinds of endothelial cell proliferation assays, namely with or without vascular endothelial growth factor (VEGF) stimulation, were used to evaluate respectively the anti-angiogenic or the pro-angiogenic activity of herbal ingredients. Only the positive results were reported. Human Umbilical Vein Endothelial Cells (HUVECs) from Cascade Biologics (Portland, USA) were cultured in endothelial cell medium (Sciencell Research Laboratory) together with 10% fetal bovine serum and endothelial cell growth supplement. This mixture was sub-cultured using a 1:2 ratio with Trypsin/EDTA solution provided by the manufacturer. Herbal ingredients were purchased from the National Institute for the Control of Pharmaceutical and Biological Products, China. HUVECs (5×10^3^ per well) in a 96-well plate were starved with 0.1% FBS medium and then treated with or without VEGF (5-10 ng/ml) along with different concentrations of herbal ingredients for 48 hours. Cell viability was determined by Cell Counting Kit (CCK-8, Dojindo, Japan) following the measurement of optical density values using MRX Revelation Absorbance Reader.

#### Herb interaction measurement of DMIM-extracted herb pairs

We investigated whether combination effects were produced by DMIM-extracted herb pairs and whether there was a role for the natural properties of the herbs. The highest single compound model [18] was used as the reference model for measuring additivity to identify herbal interactions such as synergism or antagonism. The combination effects were determined by selecting the greatest effect produced by each of the combination’s individual compounds using similar concentrations as in the combination. Positive or negative deviations from this predicted additivity demonstrated synergistic or antagonistic interactions.

### Co-module analysis for the DMIM-extracted herbal formula

#### Co-module concept, herbal formula selection and biological data preparation

To further explore the combination mechanism of DMIM-extracted herbal formulae, we propose a new concept of “co-module” based on the assumption that there may exist certain consistent and common biological patterns, which act as “co-modules”, underlying networked herbs and their targeted diseases simultaneously. We took a famous formula, “*Liu-wei-di-huang*” (LWDH, also known as Rehmannia Six, Six Ingredient Rehmannia or Rokumi-gan), as an example, since we found that all six herbs of this formula are connected closely in the DMIM-extracted herb network including Shan-zhu-yu (*Fructus Corni*), Ze-xie (*Rhizoma Alismatis*), Dan-pi (*Cortex Moutan*), Di-huang (*Radix Rehmaniae*), Fu-ling (*Poria Cocos*) and Shan-yao (*Rhizoma Dioscoreae*). Then, we collected the biological entities (genes or gene products) affected by individual herbs (compounds) of LWDH from PubMed and China National Knowledge Infrastructure (http://www.cnki.net). This resulted in a total of 146 manually collected genes or gene products, called LWDH genes, contributed respectively to the actions of the LWDH constituent six herbs. 127 LWDH genes were nodes of the protein-protein interaction (PPI) network (HPRD, release 7). Next, we collected the documented diseases for which the LWDH formula may serve as a potential treatment. This resulted in 16 diseases containing 9 types of cancer (Prostate cancer, Melanoma, Stomach cancer, Breast cancer, Esophageal cancer, Lung cancer, Hepatocellular carcinoma, Multiple myeloma and Leukemia), 5 diseases with dysfunction of the neuro-endocrine-immune-metabolism system (Parkinson disease, Asthma, Allergy, Rheumatoid arthritis and Diabetes), and 2 cardiovascular disorders (Hypertension and Atherosclerosis) (see literature in **Additional file 1**). By mapping these 16 diseases into the OMIM database, we identified 73 exclusive phenotypes with OMIM IDs and obtained 224 disease genes called LWDH-disease genes, 173 of which were networked in HPRD.

#### Performing co-module analysis for LWDH and LWDH-treated diseases

We conducted the co-module analysis from the following three aspects. (1) We analyzed the enriched KEGG pathways for either LWDH genes or LWDH-disease genes with a false discovery rate less than 0.05 by Fisher Exact test in DAVID [19]. (2) We evaluated the “closeness”(average shortest path) between LWDH genes and LWDH-disease genes in the PPI network and used the permutation test to calculate the statistical significance of the average shortest path. Here we kept the original 127 LWDH genes and randomly selected other 173 disease genes from 1273 networked genes in all 3074 non-redundant disease genes stored in the OMIM *moridmap.txt* (Feb 22, 2008); this was repeated independently 2000 times. (3) We calculated the average phenotype similarity score determined by the cosine of vector angle [20] of 73 phenotypes of LWDH-diseases, and evaluated the statistical significance by comparison with randomly selected 73 OMIM phenotypes for 2000 times.

### Statistical analysis

The mutual information statistics were transformed to equivalent odds ratios using monotonic transform and then subjected to standard *χ*^2^ test. In doing so, we used *χ*^2^ test to test whether the occurrence of the two herbs in the formulae is correlated with each other by generating a contingency table. Experimental data from the *in vitro* assay were presented as mean±SD (Standard Deviation) of four independent experiments with six repeat wells for each experiment. The statistical difference between treatments was determined by the *t* test.

## Results

### DMIM-extracted herb network from Collaterals-related formulae

**Figure 1:**
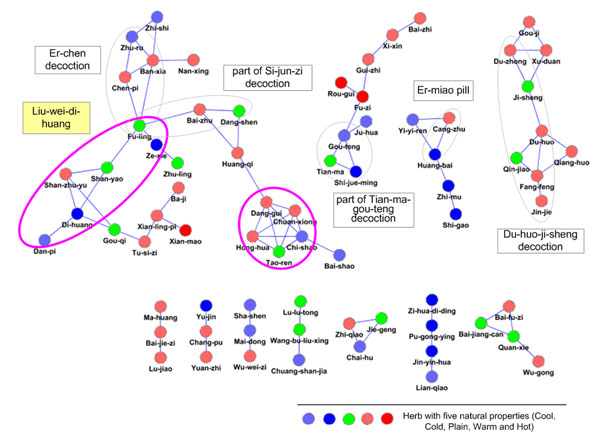
**DMIM-extracted herb network from 3865 herbal formula.**This herb network is constructed from the top 100 herb pairs extracted by DMIM. Herbs with different natural properties and six classical herbal formulae are presented in the network. Data about only two interacted herbs are not shown.

DMIM was used for extracting the combination rule of 3865 Collaterals-related formulae. In all 3865 formulae, we found that eight of the top 10 most frequently occurring herbs (Table 2) are reported to pro-angiogenesis or anti-angiogenesis activity [9,22]. This provides evidence that the Collaterals-related formulae may have a possible relationship with angiogenic disorders. Each of the top 100 DMIM-extracted herb pairs had statistical significance (P < 0.05, *x*^2^ test). Table 3 summarized the top 20 DMIM-extracted herb pairs with the highest rankings; six of these herb pairs are novel when compared with traditionally-defined herb pairs [16,17]. Interestingly, we found that Gan-cao (*Radix Rhizoma Glycyrrhizae*), a commonly-used supplementary herb (“Guide” in MASG), ranked 2nd with a frequency of 38.37% in all 3865 herbal formulae. However, the position of herb pairs containing Gan-cao fell to 195 (Table 3), suggesting that the DMIM method was able to balance the frequency, independence, and relative distance in the herbal formulae. Figure 1 shows that we constructed a herb network by using the interactions of the top 100 herb pairs extracted by DMIM, in which we found that full or part of six classical herbal formulae are nicely recovered. The distinct modular feature is also observed from the DMIM-extracted herb network.

**Table 2:** Top 10 herbs in 3865 Collaterals-related formulae

Rank	Herbs in Collaterals-related herbal formula		Natural Property	Frequency	Angiogenesis activity [9,22]
	Chinese Name	English Name			
1	Dang-gui	*Radix Angelicae Sinensis*	Warm	49.50%	Pro-angiogenesis
2	Gan-cao	*Radix Rhizoma Glycyrrhizae*	Neutral	38.37%	Anti-angiogenesis
3	Chuan-xiong	*Rhizoma Chuanxiong*	Warm	32.32%	Pro-angiogenesis
4	Chi-shao	*Radix Paeoniae Rubra*	Cool	30.79%	Pro-angiogenesis
5	Dan-shen	*Radix et Rhizoma Salviae Miltiorrhizae*	Cool	29.62%	Pro-/Anti-angiogenesis
6	Niu-xi	*Radix Achyranthis Bidentatae*	Neutral	26.39%	Unknown
7	Huang-qi	*Radix Astragali*	Warm	25.85%	Pro-angiogenesis
8	Hong-hua	*Flos Carthami*	Warm	24.94%	Unknown
9	Di-huang	*Radix Rehmanniae*	Cold	24.92%	Pro-angiogenesis
10	Bai-shao	*Radix Paeoniae Alba*	Cool	21.89%	Pro-angiogenesis

**Table 3:** Top 20 DMIM-extracted herb pairs

Rank	Herb 1			Herb 2			DMIM Score
	Chinese Name	English Name	Natural Property	Chinese Name	English Name	Natural Property	
1	Mo-yao	*Myrrh*	Neutral	Ru-xiang	*Frankincense*	Warm	2.9528
2	Hong-hua	*Flos Carthami*	Warm	Tao-ren	*Semen Persicae*	Neutral	1.8057
3	E-zhu	*Rhizoma Curcumae*	Warm	San-len	*Rhizoma Sparganii*	Neutral	0.97254
4	Long-gu	*Drgon's Bones, Fossilizid*	Neutral	Mu-li	*Concha Ostreae*	Cool	0.78572
5	Da-zao	*Fructus Jujubae*	Warm	Shen-jiang	*Rhizoma Zingiberis Recens*	Warm	0.63477
6	Quan-xie	*Scorpio*	Neutral	Wu-gong	*Scolopendra*	Warm	0.56607
7	Du-huo	*Radix Angelicae Pubescentis*	Warm	Qiang-huo	*Rhizoma et Radix Notopterygii*	Warm	0.45846
8	Cang-zhu	*Rhizoma Atractylodis*	Warm	Huang-bai	*Cortex Phellodendri*	Cold	0.43511
9	Pu-huang	*Pollen Typhae*	Neutral	Wu-ling-zhi	*Trogopterus Dung*	Warm	0.42285
10	Bai-zhu	*Rhizoma Atractylodis Macrocephalae*	Warm	Fu-ling	*Poria*	Neutral	0.41082
11	Shi-chang-pu	*Rhizoma Acori Tatarinowii*	Warm	Yuan-zhi	*Radix Polygalae*	Warm	0.40238
12	Chi-shao	*Radix Paeoniae Rubra*	Cool	Tao-ren	*Semen Persicae*	Neutral	0.39792
13*	Jiang-can	*Bombyx Batryticatus*	Neutral	Quan-xie	*Scorpio*	Neutral	0.3707
14	Ban-xia	*Rhizoma Pinelliae*	Warm	Chen-pi	*Pericarpium Citri Reticulatae*	Warm	0.34629
15*	Dang-shen	*Radix Codonopsis*	Neutral	Huang-qi	*Radix Astragali*	Warm	0.34084
16*	Chuan-xiong	*Rhizoma Chuanxiong*	Warm	Hong-hua	*Flos Carthami*	Warm	0.32943
17*	Chuan-xiong	*Rhizoma Chuanxiong*	Warm	Tao-ren	*Semen Persicae*	Neutral	0.31513
18	Chuan-xiong	*Rhizoma Chuanxiong*	Warm	Dang-gui	*Radix Angelicae Sinensis*	Warm	0.29861
19*	Chi-shao	*Radix Paeoniae Rubra*	Cool	Hong-hua	*Flos Carthami*	Warm	0.29351
20*	Bai-zhu	*Rhizoma Atractylodis Macrocephalae*	Warm	Dang-shen	*Radix Codonopsis*	Neutral	0.29154
195	Bai-shao	*Radix Paeoniae Alba*	Cool	Gan-cao	*Radix Rhizoma Glycyrrhizae*	Neutral	0.052203

*: Herb pairs extracted by DMIM but not recorded in traditionally-defined herb pairs.

### Measurement of angiogenic activities for DMIM-extracted modular herbs

**Figure 2:**
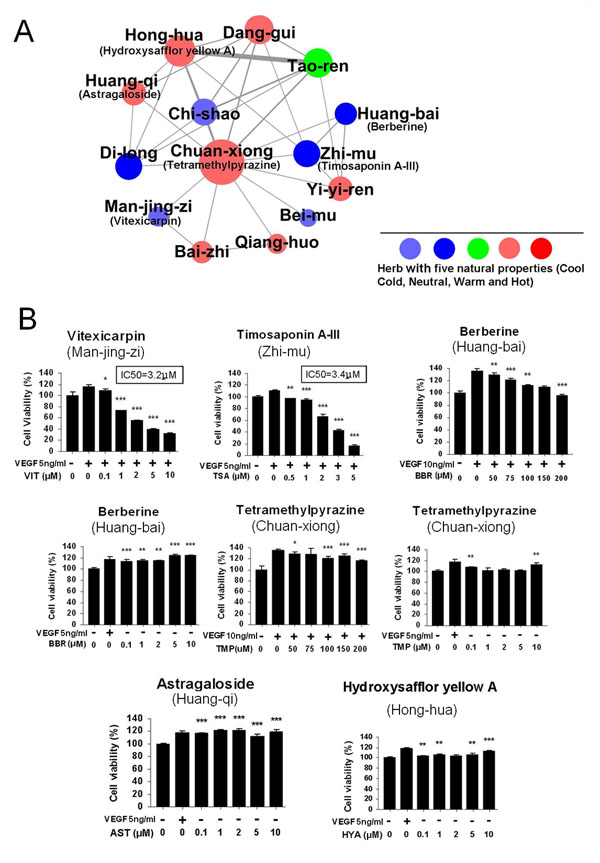
**Angiogenic activities of major ingredients in DMIM-extracted herbs. **A. The extended hub module in DMIM-extracted herb network. Each node corresponds to a herb colored according to their natural properties. The size of each node is proportional to the number of herbs connecting to it. A solid line links a herb pair while the width of the lines is proportional to the DMIM score. B. Experimental results of the herb ingredients in the module. The pro- or anti-angiogenic effects of each herb were delineated by pro- or anti-angiogenic screening model respectively.

As shown in Figure 1, the hub module or the interconnected sub-network in the DMIM-extracted herb network is centered on the most frequently occurring herbs, Chuan-xiong (*Rhizoma Chuanxiong*) and Dang-gui (*Radix Angelicae Sinensis*). We extended this hub module to all herb pairs with statistical significance (χ^2^ test, P<0.05) (Figure 2A) and assumed that herbs presented in this module could have potential angiogenic activities. By selecting the major herbal ingredients in these herbs and taking their natural properties into consideration, the following *in vitro* experimental results support our hypothesis. As shown in Figure 2B, in the hub module, Vitexicarpin (VIT) and Timosaponin A-III (TSA) as major ingredients taken from two herbs with Cold properties were very active on inhibiting endothelial cell proliferation (IC50_VIT_=3.2μM; IC50_TSA_=3.4μM respectively). Also, Hydroxysafflor yellow A (HYA) and Astragaloside (AST) from herbs with Hot properties had partial pro-angiogenesis activities when compared with the VEGF treatment group. Another trend (Figure 2B) was that Berberine from *Huang-bai* (*Cortex Phellodendri*) and Tetramethylpyrazine (TMP) from *Chuan-xiong* had a biphasic effect on endothelial cells proliferation. Lower doses caused an increase in cell proliferation whereas higher doses resulted in an anti-angiogenic response. Overall, the experimental results validated the potential angiogenic activities of the modular herbs.

### Measurement of DMIM-extracted modular herb interactions

**Figure 3:**
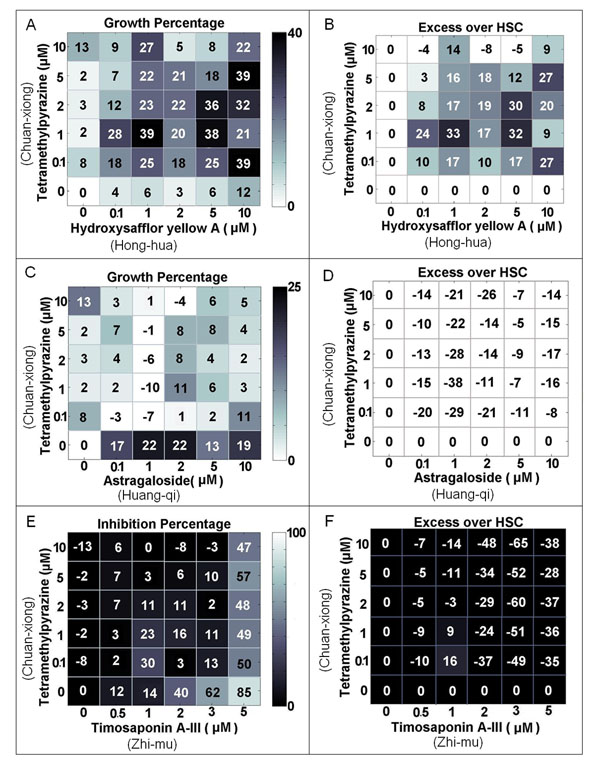
**Combination effects in DMIM-extracted herb pairs.**According to the HSC model, the dose matrix indicates the combined response of TMP in combination with three other compounds at six different doses. The color of the gird denotes the level of cell growth stimulation or inhibition. The growth percentage and inhibition percentage were calculated by the pro- or anti-angiogenic screening model respectively (A, C and E). B, D and F show the calculated excess growth or inhibition percentage over the HSC additivity model. The percentage above or below zero denotes the combination with synergism or antagonism, respectively.

We evaluated whether modular herbs with different properties had potential combination effects. Figure 3 shows that HUVECs were treated with different compound combinations in a 6×6 dose matrix using the same conditions as the cell growth assay. By using the highest single compound model [18] we found that TMP (from Chuan-xiong with Warm properties) in combination with HYA (from Hong-hua with Warm properties) caused moderate synergistic pro-angiogenic activity, whereas antagonistic effects were observed when TMP was combined with AST (from Huang-qi with Warm properties). Noticeably, TMP and TSA (from Zhi-mu with Cool properties) produced obvious antagonism at higher concentrations (Figure 3). We also observed that the traditional herb pairs Chuan-xiong and Huang-qi, and the novel herb pairs Chuan-xiong and Hong-hua identified by DMIM exhibited clear combination effects on endothelial cell proliferation. These results suggest that the different interaction patterns of herb pairs may be associated with their different herb properties, although this association remains unclear.

### Co-module underlying DMIM-extracted herbal formula in treating different diseases

**Figure 4:**
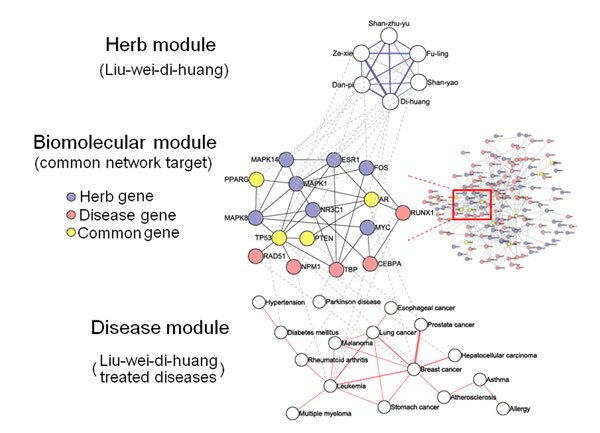
**The co-module underlying *Liu-wei-di-huang* formula and diseases.** For the herb module, two herbs from the *Liu-wei-di-huang* are linked if they have common responsive genes. For the disease module, two diseases are linked if they have common disease genes. The width of the solid lines is scaled with the number of common herb or disease genes. All herb genes and disease genes are mapped to the protein-protein interaction network. A biomolecular module as a common network target and associated with both the herb module and the disease module is extracted with dashed lines.

DMIM can recover and connect all six herbs of the *Liu-wei-di-huang* formula. This formula is reported to potentially treat 16 types of diseases (**Additional file 1**). Thus, we performed a co-module analysis to explore the potential combination mechanism of DMIM-extracted herbal formula. Table 4 shows that LWDH genes as well as LWDH-disease genes are mainly enriched in cancer pathways and neuro-endocrine-immune pathways (see **Additional file 2** for detailed statistics). Moreover, based on the PPI network, it is noted that the average shortest path length is significantly smaller between LWDH genes and LWDH-disease genes than between LWDH genes and randomly selected disease genes (P<0.0001, 2000 permutations). This highlights the specificity of the LWDH for treating these 16 different diseases. In addition to this, the average phenotype similarity scores for these 16 LWDH diseases are higher than the scores of random controls (P=0.0248, 2000 permutations), suggesting that it might be possible to group together LWDH-treated diseases through a common molecular basis. These findings evidenced that LWDH might act on a common network target underlying these diseases, and we can capture the “one formula, different diseases” relationship from a co-module viewpoint based on multilayer networks of herb-biomolecule-disease (Figure 4).

**Table 4:** Enriched pathways of *Liu-wei-di-huang* genes and *Liu-wei-di-huang*-treated disease genes identified by DAVID

Category	Enriched KEGG pathway	False discovery rate
*Liu-wei-di-huang* genes	hsa05200:Pathways in cancer	1.82E-06
	hsa05014:Amyotrophic lateral sclerosis	3.82E-06
	hsa04620:Toll-like receptor signaling pathway	6.42E-06
	hsa04660:T cell receptor signaling pathway	1.56E-05
	hsa04210:Apoptosis	8.13E-04
	hsa04621:NOD-like receptor signaling pathway	0.002737
	hsa05222:Small cell lung cancer	0.00471
	hsa04080:Neuroactive ligand-receptor interaction	0.007635
	hsa05210:Colorectal cancer	0.03486
*Liu-wei-di-huang*-treated	hsa05200:Pathways in cancer	2.35E-12
disease genes	hsa05210:Colorectal cancer	1.11E-07
	hsa05221:Acute myeloid leukemia	2.58E-07
	hsa05213:Endometrial cancer	5.92E-07
	hsa05220:Chronic myeloid leukemia	9.79E-05
	hsa05215:Prostate cancer	1.38E-04
	hsa04930:Type II diabetes mellitus	2.25E-04
	hsa05218:Melanoma	3.68E-04
	hsa05216:Thyroid cancer	0.002937
	hsa05214:Glioma	0.005064
	hsa05223:Non-small cell lung cancer	0.008102
	hsa04950:Maturity onset diabetes of the young	0.01155
	hsa05212:Pancreatic cancer	0.019292

## Discussion

In this work, we proposed a distance-based mutual information model, DMIM, to uncover the combination rule embedded in herbal formulae, which not only uses mutual information entropy but also introduces a new factor, “between-herb-distance”, into measuring the tendency of two herbs to form an herb pair. This makes DMIM suitable for deciphering herbal formulae and distinguishes it from other analytical methods such as clustering. For example, herb_1_ and herb_2_ are often used together to reduce toxicity and side-effects, while herb_2_ and herb_3_ may be clustered into a single category because of their co-location in similar organs or meridians. According to the principles of clustering, herb_1_, herb_2_ and herb_3_ may be clustered into one category, but in reality, herb_1_ and herb_3_ have no inherent relationship. Moreover, the results of clustering are qualitative rather than quantitative and clustering does not show which herbs have a tendency to form herb pairs. DMIM avoids these pitfalls by calculating the mutual information entropy for each of the herbs and their “between-herb-distance”.

We demonstrated the reliability and usefulness of DMIM by using 3865 Collaterals-related herbal formulae. Firstly, we showed that the DMIM method retains the traditional combination rule of TCM. DMIM identified many herbal pairs which have already been defined (Table 3). We also found that the DMIM-extracted herb network identified six classical herbal formulae from the paired herbs (Figure 1), which are expressed as connected sub-networks. On the other hand, DMIM-extracted herb network can eliminate the disturbance from herbs such as Gan-cao (*Radix Rhizoma Glycyrrhizae*), a widely used “Guide” herb that coordinates the actions of other herbs in formulae, though it ranks at top 2 in 3865 herbal formulae.

Next, we showed that DMIM has the potential to discover angiogenic herbs and non-addictive herb pairs from TCM. This study found that the 10 most common herbs in the 3865 formulae had potential angiogenic effects (Table 2) [9,22]. We also conducted *in vitro* assay to evaluate the extended hub module for Chuan-xiong (*Rhizoma Chuanxiong*) and Dang-gui (*Radix Angelicae Sinensis*) in the DMIM-extracted herb network (Figure 2A). As the ingredients of herbs are very complicated and the quality of herbs is still unstable, for simplify, this work used major ingredients of herbs to perform experiments. Results showed that the herbs or herb pairs in the hub modules produced anti-angiogenic or pro-angiogenic activities, suggesting that the modular herbs may have functional dependence. In particular, we detected the novel bioactivity of two herb ingredients which inhibited angiogenesis, including Vitexicarpin (IC50 = 3.2 μM) and Timosaponin A-III (IC50 = 3.4 μM) (Figure 2B). We also validated the synergistic effects produced by DMIM-extracted novel herb pairs such as Chuan-xiong and Hong-hua (Table 3 and Figure 3).

Additionally, in this study, we observed that the active compounds from the herbs with different natural properties might account for their different angiogenic responses (Figure 2B). For instance, major ingredients from Cool/Cold herbs tend to produce anti-angiogenic activities whereas major ingredients from Warm/Hot herbs tend to exert pro-angiogenic activities [22]. The dose-response relationship is another way to understand the characteristics of the herb’s natural properties. We found that Berberine and Tetramethylpyrazine can cause a pro-angiogenic effect at low dose and anti-angiogenic effects at high dose (Figure 2B), suggesting that some herbs may cause biphasic regulation if different dosing regimens are used. For herb interaction effects we assumed that herb pairs in a formula with the same properties were more likely to lead to synergistic interactions, whereas combinations with different properties were inclined to cause antagonism. As shown in Figure 3, combination effects from herb pairs with the same properties (e.g. Chuan-xiong and Hong-hua) and different properties (e.g. Chuan-xiong and Zhi-mu) support our assumption, but the combination of Chuan-xiong and Huang-qi is not the case, making it an open question whether or not herbal properties are related to herb combination behaviours. 

Last but not least, we demonstrated that the DMIM-extracted herbal formula, *Liu-wei-di-huang*, may have its molecular basis for treating different diseases in a co-module manner (Figure 4). LWDH is one of the most famous TCM formulae developed during the Song dynasty in China. Results show that the six herbs in LWDH not only have high DMIM scores, but also connected closely with common responsive genes enriched in cancer pathways and neuro-endocrine-immune pathways (Table 4). Interestingly, LWDH genes show a significantly close relationship with LWDH-disease genes in the PPI network (P<0.0001), forming a co-module underlying herbal formula as well as different diseases. Moreover, the 16 LWDH-treated diseases mainly including cancer, neuro-endocrine-immune-metabolism, and cardiovascular disorders show high phenotype similarity scores (P=0.0248) and might share a overlapped molecular basis associated with the angiogenic processes as well as the imbalance of the human body [23,24]. Such phenomena of “one formula, different diseases” reinforce the idea that different diseases with similar phenotypes might possess internal coherence [25-27], and a group of diseases with similar mechanisms might be able to be treated by intervening their common network target [28,29], which in turn illustrates the rationality of multicomponent therapies such as herbal formulae (Figure 4). The novel concept of co-module throughout the multilayer networks of herb-biomolecule-diseases may promote our awareness of herbal formulae as well as multicomponent therapies.

DMIM is currently the first step towards building herb network from TCM herbal formula. For future work, DMIM could be generalized to mine synergistic combinations made up of more than two herbs by replacing the “between-herb-distance” with a properly defined index of the distance among multiple herbs in a formula or by introducing multivariate mutual information. As this work treats formula independently, we will take the redundancies and correlations between formulae into consideration for calculating the herb distance. The dose information and natural properties of herbs (as measures of interaction) are also the next step to create a multi-weight herb network. Moreover, we believe that the combination mechanism of herbal formulae will be more deeply identified in a “co-module” manner and contribute to the progression of the modern TCM as well as network pharmacological studies [13].

## Conclusions

DMIM yields a systematic framework for scoring herb pairs and the resulted herb network can uncover some combination rules of TCM. We also provide preliminary clues that the “co-module” across multilayer networks of herb-biomolecule-disease may be responsible for the combination mechanism underlying herbal formulae. This study is the first step forward in exploring the unique theories of TCM herbal formula by network biology approaches and may also benefit the coming network pharmacology as well.

## Competing interests

The authors declare that they have no competing interests.

## Authors' contributions

SL conceived and designed the experiments, analyzed the data and wrote manuscript. BZ participated in the cell experiments and writing manuscript. YW, DJ and NZ participated in the computational works and writing manuscript.
